# Fetal and infant mortality of congenital syphilis reported to the Health Information System

**DOI:** 10.1371/journal.pone.0209906

**Published:** 2019-01-04

**Authors:** Surama Valena Elarrat Canto, Maria Alix Leite Araújo, Angélica Espinosa Miranda, Ana Rita Paulo Cardoso, Rosa Lívia Freitas de Almeida

**Affiliations:** 1 Department of Health Surveillance, Ceará State Secretary of Health, Fortaleza, Ceará, Brazil; 2 Collective Health Post Graduation Program, University of Fortaleza, Fortaleza, Ceará, Brazil; 3 Department of Social Medicine, Federal University of Espírito Santo, Vitória, Espírito Santo, Brazil; Leibniz Institute for Prevention Research and Epidemiology BIPS, GERMANY

## Abstract

**Background:**

Congenital syphilis (CS) is a major cause of mortality in several countries, especially in Latin America and the Caribbean. This study aimed to analyze fetal and infant mortality of CS reported to the Health Information System in a State in Northeastern Brazil.

**Methods and results:**

This was a cross-sectional study that analyzed the deaths of CS from 2010 to 2014 through the linkage of the Mortality Information System (SIM) and the Notifiable Diseases Information System (Sinan). The Statistical Package for the Social Sciences (SPSS) version 23.0 was used to calculate the rates of Fetal, Perinatal, Neonatal (early and late), and Postneonatal Mortality. Simple linear regression was performed. Fisher's exact test or Pearson's chi-square test were used for comparison of proportions and Student's t-test was used for comparison of means.

Of the 414 cases reported to the SIM as deaths possibly caused by CS, 44 (10.6%) presented CS as the underlying cause. From 2010 to 2014 the Infant Mortality Rate of CS was 16.3 per 100,000 live births (y = 0.65x + 14.33, R^2^ = 0.2338, p = 0.003). There was an 89.4% underreporting of deaths. Perinatal deaths and fetal deaths of CS accounted for 87.7% and 73.9% of total deaths, respectively.

**Conclusions:**

The results of the study revealed a significant Fetal and Infant Mortality rate of CS and demonstrated the importance of using the linkage method in studies that involve the analysis of secondary data obtained from mortality and disease reporting systems. The underreporting of CS as a cause of fetal and infant mortality leads to unawareness of the reality of deaths from this disease, hindering the development of public policies aimed at its prevention.

## Introduction

Congenital syphilis (CS), a systemic infection that occurs when the pregnant woman infected with *treponema pallidum* transmits the bacterium to the baby, is a major cause of mortality in several countries [1,2], especially in Latin America and the Caribbean [3]. In 2008, an estimated 1 360 485 pregnant women had active syphilis worldwide. This situation can cause serious consequences for the baby, such as stillbirth, neonatal mortality, prematurity and low birth weight [1].

In 2010, the World Health Organization/Pan American Health Organization (WHO/PAHO) approved the Plan of Action for the Elimination of Mother-to-Child Transmission of HIV and Syphilis in the Americas, which proposed to reduce the incidence of CS to 0.5 cases per 1000 live births or less by the year 2015 [4]. However, few countries have achieved this goal [5], which demonstrates how difficult it is to control the infection. In Brazil, despite the good prenatal care coverage, such goal has not been achieved, possibly because of the poor quality of prenatal care [6]. CS mortality rate in 2015 was 7.4 per 100,000 live births (LB) [7].

To control infection in the country, pregnant women should be tested for syphilis twice during prenatal care and treatment should be offered to infected women and their sexual partners [8]. However, despite all efforts and a 89.9% rate of prenatal care coverage in the country [6] CS remains a major public health problem. Estimates show high incidence and mortality rates, which may be related to the failure to diagnose or the delay in the diagnosis of syphilis and the failure to treat the pregnant woman or even her inadequate treatment [6,9,10].

Surveillance, monitoring and assessment of all diagnosed cases are important strategies that may support the elimination of CS. In this regard, the WHO has developed indicators that facilitate comparability across countries in terms of outcome (assuming that the same case definition is used), but does not capture and articulate the complexity of individual health systems [11]. In Latin America and the Caribbean, these indicators are obtained from the Perinatal Information System (PIS). In Brazil, they can be found in the Health Information System (*Sistema de Informação em Saúde–SIS*).

The SIS is a group of systems that contain standardized instruments for collecting information on diseases, injuries and deaths that represent major public health problems. The Notifiable Diseases Information System (*Sistema de Informação de Agravos de Notificação–Sinan*), the Mortality Information System (*Sistema de Informação sobre Mortalidae–SIM*), the Live Births Information System (*Sistema de Informações sobre Nascidos Vivos–Sinasc*) and the Hospital Information System (*Sistema de Informações Hospitalares–SIH*) are all part of the SIS and reporting to these systems is mandatory [12].

Therefore, this study aimed to analyze fetal and infant deaths to CS reported to the SIM and to the Sinan and to identify which codes of the International Classification of Diseases and Related Health Problems (ICD-10) have predominated in the death certificates of underreported cases of CS in a state in Northeastern Brazil. It is believed that the analysis of different systems can contribute to the identification of the real magnitude of fetal and infant deaths from CS and to the development of prevention and control measures.

## Materials and methods

This is a cross-sectional study of fetal and infant deaths of CS in Ceará, the fourth largest state in Northeastern Brazil and the 17^**th**^ Brazilian state in terms of total area. Ceará has a population of 8 452 381 inhabitants, representing 4.43% of Brazil’s population and 15.9% of the population of the Northeast Region [13].

Data were collected from different databases (SIM and Sinan) to maximize the reliability of the results and to minimize underreporting of cases. Diagnosed cases of syphilis in pregnant women and CS are reported to the Sinan and deaths–from syphilis or other diseases–are reported to the SIM. In this research, we used the record linkage method, a relatively low-cost and feasible method that allows more detailed analyses of the quality of official data [14,15].

All infant and fetal deaths reported to the SIM and all cases of CS and syphilis in pregnancy (SP) reported to the Sinan from January 1, 2010 to December 31, 2014 were included. Cases of people living outside the State of Ceará, duplicates, corrupted files and infant deaths from external causes were excluded.

The information reported to the SIM is originated from Death Certificates (DC) which must be completed and issued by a physician. The SIM is an online system that allows information to be viewed by the State Government departments and agencies and the Ministry of Health immediately after it is entered online. The State Government is responsible for the monitoring and analysis of data processed by City Governments [16].

The Sinan receives reports of cases of diseases, injuries and public health events included in the National Compulsory Reporting List which are recorded in specific sections. Cases of SP and CS are reported to this system [17] based on case definitions used by the Brazilian Ministry of Health [7]. The information relating to each of these systems is manually input into specific records in the city where the incident occurs. After that, the data are entered online and sent to the State Secretariats, which send them to the Brazilian Ministry of Health [12].

The data collection process involved the following steps: identification and selection of all fetal and infant deaths–regardless of ICD-10 code–reported to the SIM and of cases of syphilis in pregnancy and congenital syphilis reported to the Sinan followed by the export of the data to a single file built in 2010 Microsoft Office Excel. Databases (SIM and Sinan) were identified using different colors. During this process, the databases were merged and the mothers’ names were sorted alphabetically. In addition, typing errors were minimized and duplicates and corrupted files were removed.

After that, the records in the databases were linked using the record linkage method. We used a deterministic record linkage, which allows the manual review of record pairs that agree or disagree on identifiers in different databases. The following matching variables were considered: mother’s name, date of death, baby’s date of birth, city of residence, address and mother’s date of birth. These variables were also used to identify homonyms or similar names.

After data cleaning and standardization, the SIM data were divided into two groups: the group of fetal and infant deaths identified with ICD-10 codes for CS (A50.0-A50.9) and the group of fetal and infant deaths identified with other ICD-10 codes. Thereafter, each case was paired with the Sinan data using the mother and/or child reporting forms. This procedure aimed to identify deaths from CS reported to the SIM and check whether the cases of children and/or mothers with syphilis had also been reported to Sinan. After that, we carried out an inverse procedure, i.e., the cases of mothers and children with syphilis reported to the Sinan were identified and then we checked whether they had been reported to the SIM. Thus, our study considered deaths from CS all those identified with an ICD-10 code for CS reported to the SIM as well as those identified with other ICD-10 codes reported to the SIM but that referred to cases of children and/or mothers with syphilis reported to the Sinan.

Trend analysis was carried out using simple linear regression to determine the slope and the coefficient of determination of deaths in the analyzed period. The variables were described using proportional frequency distribution and tests were used to compare proportions (Fisher’s exact or Pearson’s chi-square) and means (Student’s t). After pairing the databases in Excel, we transferred the data to the Statistical Package for the Social Sciences (SPSS) version 23.0 for analysis.

Rates of infant deaths of CS (deaths of children less than one year of age), fetal deaths (fetal deaths after 22 weeks of pregnancy and/or birth weight of 500 grams or more), perinatal mortality (fetal and infant deaths that occurred from 22 weeks of pregnancy to the seventh day of life), early neonatal mortality (children aged 0 to 6 days old), late neonatal mortality (children aged 7 to 27 days of life) and postneonatal mortality (children aged 28 to 364 days of life) were estimated [18].

We reviewed the DC to identify cases of children with syphilis reported to the Sinan that had been reported to the SIM using ICD-10 codes other than those used for CS. Maternal variables (mother’s age; education; childbirth-related death and gestational age) and infant variables (gender and fetal weight) were assessed.

The study was approved by the Research Ethics Committee of the University of Fortaleza (UNIFOR), being Opinion No. 1.432.149/2016. Since this is a study that uses secondary databases that contain information collected routinely by professionals delivering health care and childbirth care, there was no need to obtain written consent. The data were collected from the Mortality Information System and the Notifiable Diseases Information System databases. The head of the Health Department gave written authorization to collect and use the data available in the information systems. The records of the study were kept private according to the stipulations of the law, therefore no mother or child identification could be divulged.

## Results

From 2010 to 2014, were reported to the SIM 16 184 deaths (7 856 fetal deaths and 8 328 infant deaths) and to the Sinan 5 133 cases of CS and 3 278 cases of syphilis in pregnancy.

**Fig 1:**
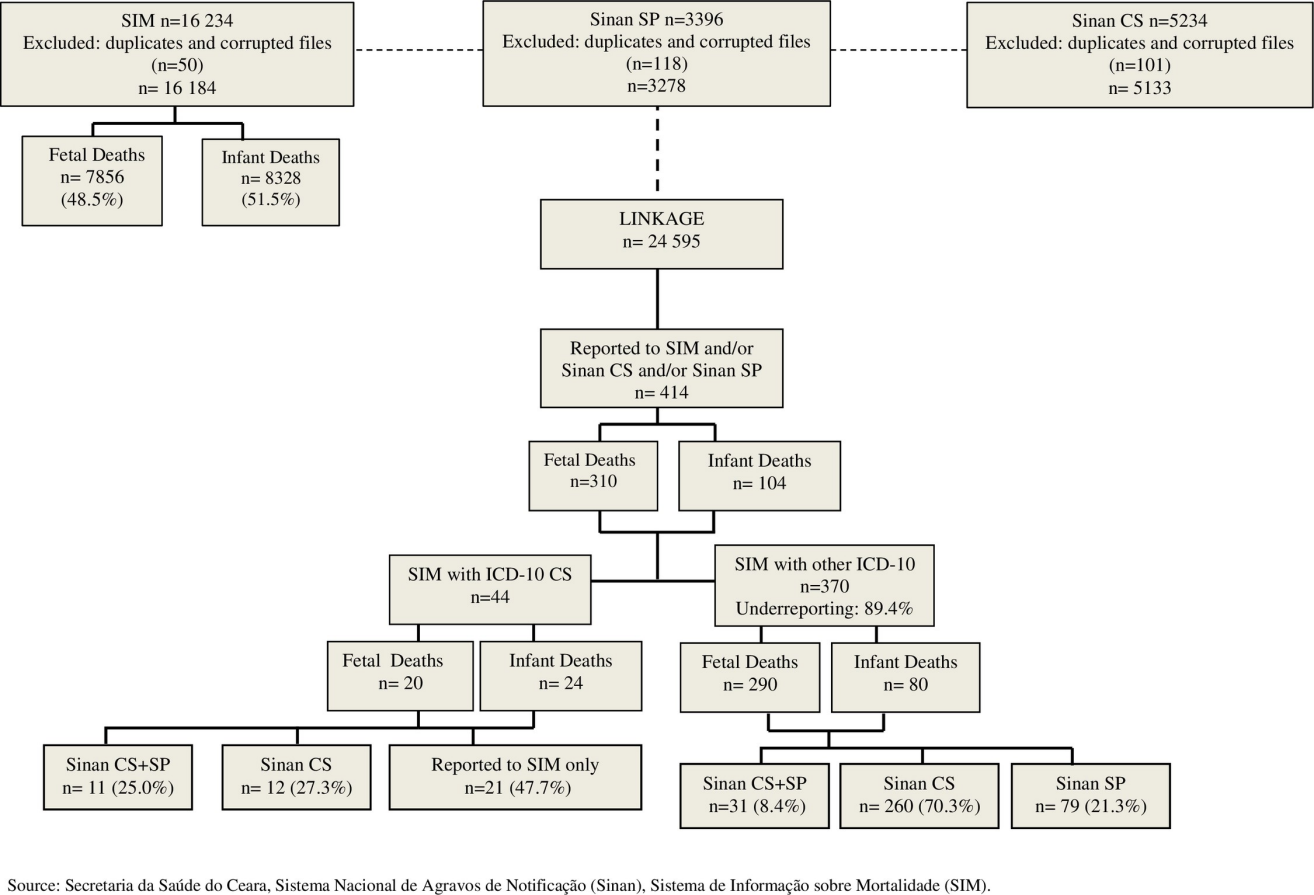
Deterministic record linkage between the Sistema de informação sobre mortalidade (SIM), Sistema de Informação de Agravos de Notificação (Sinan) Congenital Syphilis and the Sinan Syphilis in Pregnancy databases. Ceará, Brazil, 2010–2014.

CS was the underlying or contributing cause of death in 44 (10.6%) cases (20 fetal deaths and 24 infant deaths) reported to the SIM. After linkage of databases, we identified another 370 records of deaths that did not present CS as the cause of death in any of the DC sections, but that referred to cases of mothers and/or children with syphilis that had been reported to the Sinan (Fig 1).

The most common ICD-10 codes found in the records of deaths potentially caused by CS were those included in chapter XVI—Certain conditions originating in the perinatal period (P00-P96), which corresponded to 90.5% of the ICD codes found. ICD codes P90—P96 (Other disorders originating in the perinatal period) predominated in 116 records and the most common ICD code found was P95 (fetal death of unspecified cause), followed by the P00-P04 block (Fetus and newborn affected by maternal factors and by complications of pregnancy, labor and delivery) in 103 records, particularly code P00.2 (Fetus and newborn affected by maternal infectious and parasitic diseases); 98 records exhibited the P20-P29 block (Respiratory and cardiovascular disorders specific to the perinatal period), with codes P20.0 (Intrauterine hypoxia) and P29 (Intrauterine hypoxia, unspecified). Extreme immaturity, extremely low birth weight, disseminated intravascular coagulation of fetus and newborn, hydrops fetalis and Sepsis (Chapter I) were also found.

The codes described in chapter XVII—Condition originating in the perinatal period, unspecified (Q00-Q99) corresponded to 4.6% of the ICD-10 codes found and were mainly Congenital malformations of the circulatory system and Other congenital malformations

The linkage of the databases resulted in a match rate of 95.0%. After linkage, we found an underreporting of deaths from CS of 89.4%.

**Fig 2:**
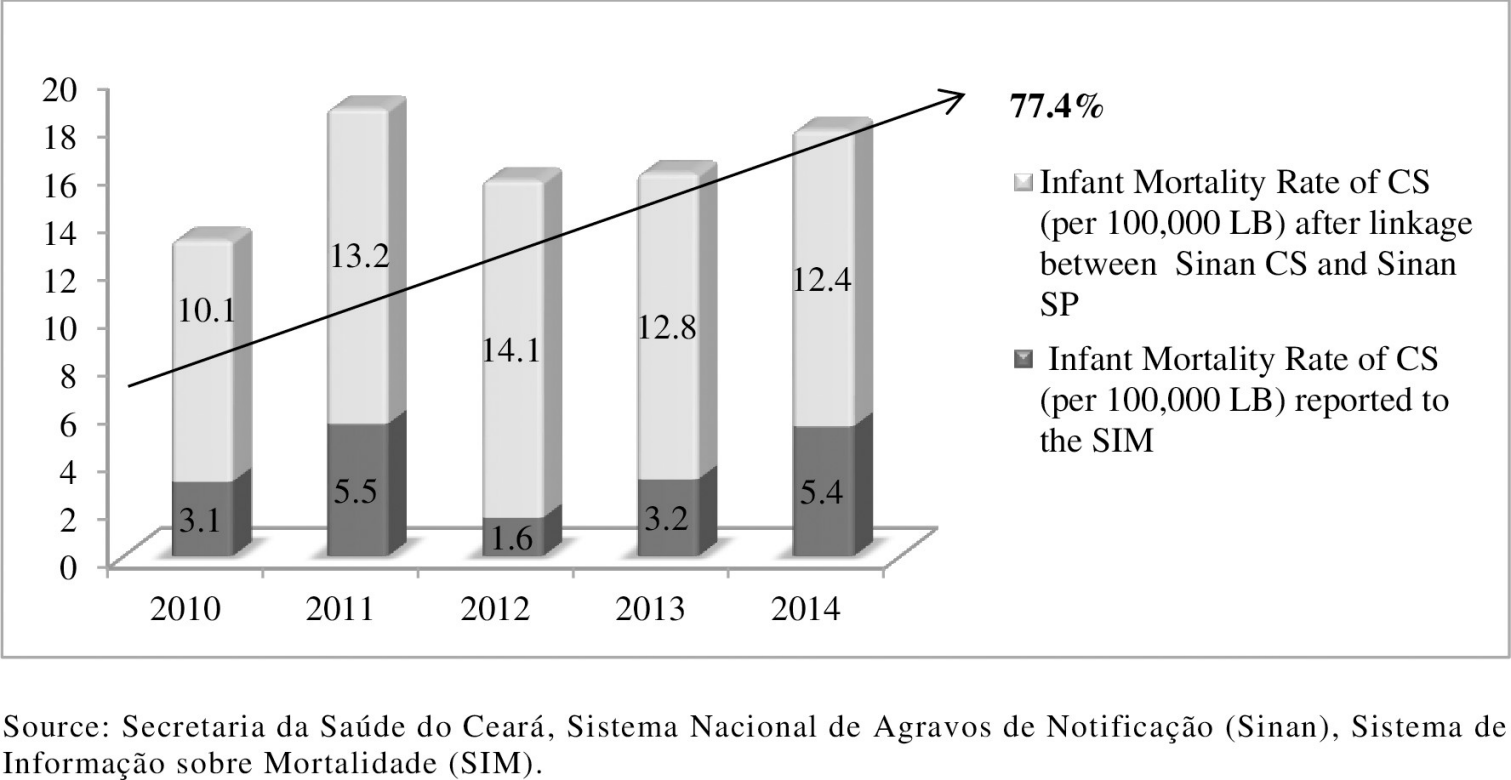
Infant Mortality rate of Congenital Syphilis reported to the sistema de informação sobre mortalidade (SIM) and after linkage between the Sistema de Informação de Agravos de Notificação (Sinan) congenital syphilis and the sinan syphilis in pregnancy. Ceará, Brazil, 2010–2014.

Fig 2 presents an analysis of the infant mortality rate of CS before and after record linkage. All the years exhibited an expressive underreporting of deaths, with a 77.4% increase over the analyzed period. Before linkage, when only deaths reported to the SIM were analyzed, the infant mortality rate of CS was 3.8 per 100, 000 LB (SD±1.8). This rate was well below that found after linkage: 12.5 per 100, 000 LB (p<0.001).

**Fig 3:**
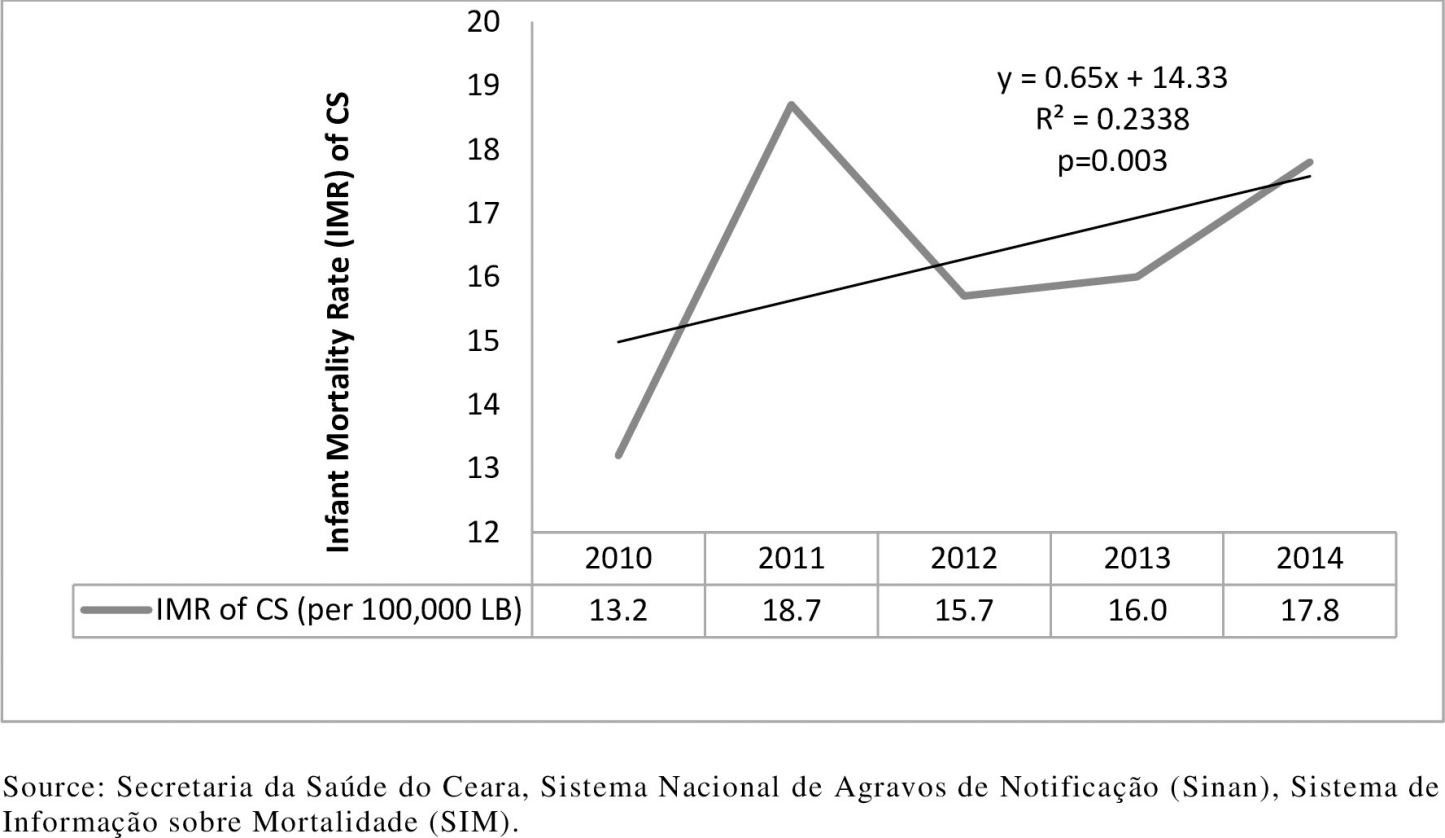
Infant Mortality rate of congenital syphilis after linkage. Ceará, Brazil, 2010–2014.

Fig 3 shows the infant mortality rate of CS after linkage. There was a systematic growth of 0.65 per year (R^2^ = 0.23; p = 0.003) over the years.

**Fig 4:**
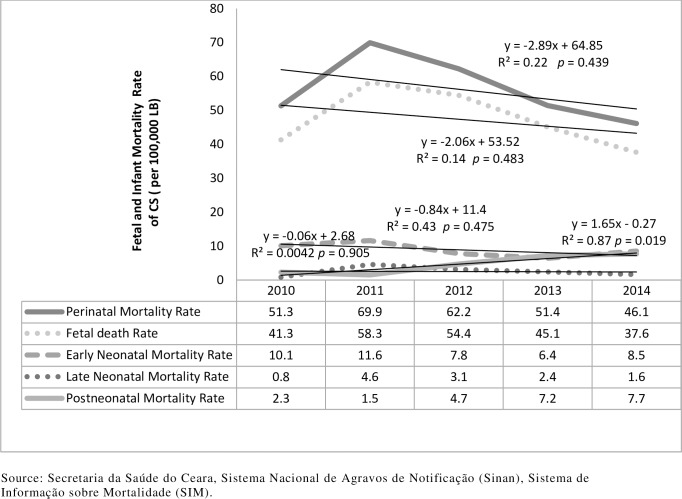
The fetal, perinatal, neonatal (early and late) and postneonatal mortality rate of congenital syphilis. Ceará, Brazil, 2010–2014.

In all, there were 104 (25.1%) infant deaths: 71% of them were neonatal deaths and 29.0% were postneonatal deaths. Early neonatal deaths accounted for 77% of all neonatal deaths. The analysis of the total (414) of fetal and infant deaths (Fig 4) revealed a mean fetal mortality rate of 47.3 per 100, 000 LB over the five years analyzed, with a decrease of 2.06 (R^2^ = 0.13). The perinatal mortality rate was 56.2 per 100, 000 LB, with a decrease of 2.89 (R^2^ = 0.22, p = 0.439). Neonatal mortality (early and late) exhibited a decrease of 0.84 and postneonatal mortality increased by 1.65 over the years, with a rate of 4.7 per 100, 000 LB (R^2^ = 0.87, p = 0.019).

Table 1 depicts information on the mother, the birth and the fetal and infant deaths. The age of the mothers ranged 12 to 42 years (mean of 24.3; SD ±6.7). In all, 57.8% of the mothers were aged 19 to 29 years and 21.7% were aged less than 18 years. There were 285 (83.3%) preterm births, with a birth weight of less than 2500 grams (84.3%).

**Table 1:** Analysis of the variables related to the mother, the birth and the fetal and infant deaths. Ceará, Brazil, 2010–2014.

Variables	n	%
**Mother’s age (n = 396)**		
≤ 18 years	86	21.7
19–29 years	229	57.8
≥ 30 years	81	20.5
**Mother’s education (n = 325)**		
≤ 7 years	208	64
≥ 8 years	117	36
**Gestational age (n = 342)**		
< 37 weeks	285	83.3
≥ 37 weeks	57	16.7
**Birth-related death (n = 336)**		
Before birth	246	71
During birth	5	3.8
After birth	85	20.5
**Fetal and infant Gender (n = 414)**		
Male	226	54.6
Female	188	45.4
**Fetal and infant weight (n = 383)**		
< 2500 g	323	84.3
≥ 2500 g	60	15.7

Source: Sistema de Informação sobre Mortalidade (SIM).

## Discussion

This study presents important aspects related to the discrepancy of records of CS in the SIM and the Sinan, as well as he considerable increase in deaths when analyzing more than one health information system. Infant mortality rate for this disease increased over the years analyzed, with peaks in 2011 and 2014, a situation also verified in other regions of Brazil [7]. Similar rates were found in other countries, reinforcing the idea that CS represents a global public health problem [1].

With regard to infant mortality from CS stated in the DC, there was a rate of 3.8 deaths per 100, 000 LB in Ceará. This rate is different from that reported by the Brazilian Ministry of Health, which was 2.2 per 100, 000 LB [7]. This difference may be explained by the fact that the analysis performed by the Ministry of Health included records of perinatal deaths reported to the SIM, whereas this study included late neonatal and postneonatal deaths as well.

The infant mortality rate in the state of Ceará decreased between 2010 and 2014. However, CS mortality rate increased in the same period [19], especially postneonatal infant mortality, and may be related to non-diagnosis in the maternity hospital, inadequate follow-up or abandonment of follow-up of these babies exposed to syphilis during pregnancy [20,21], resulting in non-recognition of CS as a cause of death in these cases [22].

Tackling infant mortality has become a priority in Brazil, many activities have been carried out to tackle the problem. The activities are primarily intended to improve access to health care and maternal and newborn care during the first years of life and have succeeded in reducing infant mortality rate. Despite the effort to control CS, there has been a growing CS epidemic in Brazil–particularly in Ceará –that has led to significant fetal and infant mortality rates [7].

We found high rates of fetal, perinatal and neonatal deaths, the latter accounting for more than half of infant deaths. It should be noted, however, that these figures may not represent the real problem given the underreporting of cases and the lack of records of abortions and stillbirths. Each year, untreated syphilis in pregnancy result in approximately 212, 000 fetal deaths worldwide, which can result in 25% to 40% of fetal deaths from CS [1,23].

More investment is needed to improve the quality of prenatal care because opportunities are also lost due to other vertically transmitted infections, such as HIV/AIDS [24,25].

In all, 29.2% of the 414 fetal and infant deaths found in this study referred to cases of mothers with syphilis reported to the Sinan. This finding shows that syphilis is probably not being diagnosed during prenatal care [26] or that diagnosed cases are not being reported. The elimination of CS is possible if diagnosis and treatment are instituted in a timely manner. Adequate treatment with Benzathine penicillin is able to avoid 97% of cases of vertical transmission [27]. Syphilis diagnosis and treatment are low-cost measures that can be easily offered in places with fewer resources, which can facilitate pregnant women’s and their partners’ access to early diagnosis and treatment, thus avoiding up to 26% of unfavorable outcomes [1,28].

Most of the children’s DC that did not indicate CS as the underlying or contributing cause of death exhibited ICD-10 codes from the chapter on certain conditions originating in the perinatal period. The clinical symptoms of CS may include changes in several systems, such as manifestations in the central nervous system, hematological alterations, especially thrombocytopenia and disseminated intravascular coagulation, pulmonary alterations, asphyxia, among others. In addition, the clinical manifestations of CS may be similar to those occurring in other infectious diseases that affect newborns, such as sepsis and, more rarely, septic shock [29–32]. Given that, we considered that these deaths may have been caused by CS, although it was not stated in the DC [29,30].

Symptomatic CS is more frequent in preterm infants and has been cited as a risk factor for prematurity. In addition, it has been shown to cause neonatal death and stillbirth [3,33].

Low birth weight, prematurity, pneumonia, asphyxia and congenital anomalies may increase the risk of perinatal death from CS. Researchers who described clinical cases of CS in a neonatal intensive care unit found prematurity, low birth weight, early sepsis, hepatosplenomegaly, hematological alterations, and asphyxia. All these clinical manifestations were found among the ICD codes analyzed in our study [31,33].

The linkage method is a low-cost and important tool to analyze secondary databases and systematic registers. It allows linking information from different databases, thus giving more reliable results. The high linkage rate between the databases (95.0%) analyzed in our study was similar to the rates reported in other studies that used the same method [22,34,35]. This method made it possible to identify an expressive underreporting of deaths from CS in all the years analyzed, a situation that has also been occurring in other countries [35] and Brazilian cities [10,22].

The absence of information stating that CS was the cause of death in the death certificates of children demonstrates the weakness of the information system and is a serious problem as it hides the real magnitude of the problem. It should be noted that epidemiological data are important in sensitizing managers to set a specific disease as a priority in public policies. Therefore, medical professionals should be trained and sensitized to state the cause of death in DC. That is, cases of syphilis in pregnancy and congenital syphilis should also be stated as causes of death in at least one of the sections of the DC.

A limitation of this study is that we used a secondary database, which may compromise the quality and reliability of information. However, in this specific research, which sought to analyze the fetal and infant mortality rate of CS, the use of different databases (SIM, Sinan CS and Sinan SP) contributed to minimizing such problem.

Underreporting of CS as one of the causes of fetal and infant deaths hides the magnitude of the problem and may represent a major obstacle to the development of public health policies to tackle the disease. Furthermore, deaths from CS nowadays are unacceptable. This is because CS is a totally preventable disease, provided that prevention actions are properly carried out during prenatal care. Thus, it is necessary to have zero tolerance for deaths from CS and take more vigorous action to improve syphilis prevention, diagnosis, treatment and surveillance.
