# Correction: Fetal and infant mortality of congenital syphilis reported to the Health Information System

**DOI:** 10.1371/journal.pone.0213214

**Published:** 2019-03-19

**Authors:** The PLOS ONE Staff

The initials for all authors appear incorrectly in the citation. The correct citation is: Canto SVE, Araújo MAL, Miranda AE, Cardoso ARP, Almeida RLF. (2019) Fetal and infant mortality of congenital syphilis reported to the Health Information System. PLoS ONE 14(1): e0209906. https://doi.org/10.1371/journal.pone.0209906.
